# Two distinct phenotypes of calcium oxalate stone formers could imply different long-term risks for renal function

**DOI:** 10.21203/rs.3.rs-4863593/v1

**Published:** 2024-09-02

**Authors:** James C. Williams, William S. Bowen, James E. Lingeman, Marcelino Rivera, Elaine M. Worcester, Tarek M. El-Achkar

**Affiliations:** Indiana University; Indiana University; Indiana University; Indiana University; University of Chicago; Indiana University

**Keywords:** kidney stones, nephrolithiasis, calcium oxalate, inflammation

## Abstract

Endoscopic and biopsy findings have identified two distinct phenotypes among individuals with calcium oxalate (CaOx) kidney stones. One phenotype exhibits normal renal papillae but shows interstitial mineral deposition, known as Randall’s plaque. The other phenotype presents with collecting duct plugging and a higher incidence of loss of papilla tissue mass. With Randall’s plaque, renal papilla injury involves the loss of small patches of calcified tissue (Randall’s plaque detaching with the stone), which likely results in damage to only a few nephrons. In contrast, collecting duct mineral plugs are very large, causing obstruction to tubular flow. Since each terminal collecting duct drains thousands of nephrons, ductal plugs could lead to the degeneration of many nephrons and a significant loss of renal glomeruli. New visualization techniques for immune cells in papillary biopsies have revealed that the Randall’s plaque phenotype is marked by the accumulation of macrophages around the plaque regions. In contrast, preliminary data on the plugging phenotype shows collecting duct damage with mineral plugs, increased T-lymphocytes throughout the papilla, and tubulitis, characterized by T-cell infiltration into nearby collecting duct epithelium. This suggests that while some CaOx stone formers may have some papillary inflammation but with minimal damage to nephrons, others suffer from obstruction to flow for many nephrons that may also include destructive inflammation in the renal tissue. We propose that the long-term risks for loss of renal function will be greater for CaOx stone formers with the plugging phenotype.

## Introduction

Urinary stone disease is highly prevalent, with about 10% of Americans suffering at least one stone episode [1], and risk of recurrence is high [2]. The projected annual cost of treating kidney stones is expected to soar to $4 billion by 2030 [1]. Calcium oxalate (CaOx) constitutes the primary component of the majority of kidney stones, with most of these in idiopathic CaOx stone formers, who have no disease to account for their stones [3].

Recent work has separated idiopathic CaOx stone formers according to whether they produced any stones that had grown on Randall’s plaque [4]. Endoscopic examination of those CaOx stone formers with Randall’s plaque stones showed abundant Randall’s plaque on their papillae, along with very little ductal plugging. CaOx stone formers with no stones on Randall’s plaque tended to have little plaque but abundant ductal plugging, along with larger stones and larger stone burdens.

Ductal plugs in stone formers tend to be very large (> 1 mm in diameter [5]), and, indeed, the plugs must be of significant size to be visible by endoscope. These plugs, of course, block flow in the terminal collecting ducts (duct of Bellini). Each of these ducts receives fluid from thousands of nephrons [6], so this form of obstruction has the potential to lead to significant nephron loss. Assuming a total of one million nephrons [7], and the Kriz & Kaissling estimate of 2,750 nephrons drained by a single terminal collecting duct [6], blocking only 4 terminal ducts creates obstruction for 1.1% of all nephrons. Some idiopathic CaOx stone formers have multiple ductal plugs in every papilla, so that a substantial fraction of nephrons must be subjected to the stress of obstruction in these patients.

Renal damage from urinary obstruction has long been linked to tissue inflammation [8]. Direct evidence of inflammation in individuals with kidney stones primarily stems from retrospective studies [9]. Two independent analyses of NHANES data have revealed a correlation between elevated serum levels of C-reactive protein and a lifetime history of kidney stones [10, 11]. Moreover, an extensive examination of nephrectomy specimens has shown heightened presence of pro-inflammatory M1 macrophages (CD68+) in renal tissues of stone formers, with the abundance of these macrophages correlating with mineral content in the medulla [12]. These findings align with the outcomes observed by Taguchi et al. [13], who noted increased macrophages, plasma cells, and neutrophils in papillary biopsies of stone formers, along with elevated mRNA signatures indicating immune cell activity.

Because ductal plugging leads to nephron obstruction, one might expect greater tissue inflammation in CaOx patients with ductal plugging than in those with Randall’s plaque. But the difference in tissue mineral location in these two phenotypes of CaOx stone former could also affect inflammation. Previous hypotheses regarding inflammation in kidney stone formers have focused on linking immune response to the interaction of mineral crystals with cells [14, 15]. Randall’s plaque crystals may interact with basal membrane cells of thin limb or connective tissue cells, whereas collecting duct plugs are up against the luminal membranes of collecting duct cells. Given these distinct mineral interactions with cells, one would anticipate unique expressions of immune cells between these two groups—Randall’s plaque and ductal plugging—reflecting the differential cell contact with mineral.

In this paper, we highlight observations supporting the hypothesis that inflammation in CaOx stone formers differs between patients with Randall’s plaque and those with ductal plugging. Furthermore, we speculate that long-term renal function outcomes may vary between these phenotypes of CaOx stone formers, advocating for further research in this domain. Targeted therapy tailored to specific phenotypes could potentially yield superior outcomes in reducing recurrence rates and mitigating the progression to chronic kidney disease [16].

## Methods

Patients in this study were recruited and studied as part of a larger project, which is described in more detail elsewhere [4]. All patients in the present study produced stones that were majority CaOx by volume (as measured by micro CT) and no patients were included who had any brushite in any part of the stone specimen or who had any systemic causes for their stones (no primary hyperoxaluria, bariatric surgery, etc.). Scoring of papillae for plaque, plug, or loss of papillary contour were done either by grading endoscopic video [4] or in the operating room during the procedure [17]. For ductal plugging, 0 was given for no visible plugs (yellow plaque deposits) or dilated ducts, 1 for the presence of 1–5 plugs or dilated ducts, and 2 for greater than 5. For each patient, scores from all graded papillae were averaged. For papillary pitting, 0 for no pitting, 1 for a pit that occupied just part of the papillary tip, and 2 for pitting that covered most of the tip of the papilla. For loss of papillary contour, 0 was given for normal papilla shape, 1 for some depression of the papilla, and 2 for complete flattening. For Randall’s plaque visible on the papilla, a score of 0 was given for mild plaque, 1 for moderate, and 2 for high coverage of the papilla tip by plaque. Biopsies were taken from papillae, fixed, and embedded in paraffin. Multiplexed imaging of antibodies staining for various protein markers was done using PhenoCycler (Akoya Biosciences) [18].

Statistics on patient data were carried out using Tukey-Kramer HSD test for continuous data and chi-square for categorical data (JMP Pro 17, JMP Statistical Discovery LLC, Cary, NC).

## Results and discussion

**Figure 1:**
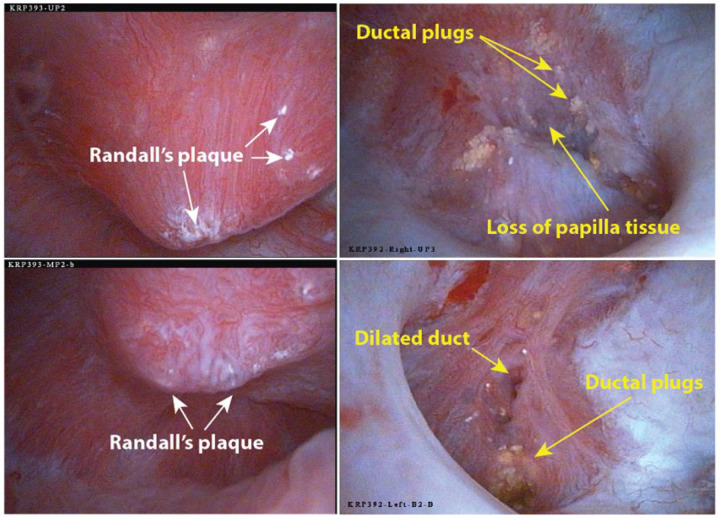
Endoscopic views of representative papillae from two patients. Both patients had majority CaOx stones and no systemic cause for their disease. The patient on the left was a 50 year-old female, and the patient on the right was a 40 year-old female. **Left**: Two papillae show normal shape, but both have some deposition of Randall’s (interstitial) plaque. **Right**: The two papillae show many plugs of mineral (yellowish in color). The upper right panel shows a papilla with a large amount of tissue loss at its tip. Lower right panel shows a compound papilla with a dilated duct. (A typical human papilla is about 1 cm wide at its base.) [add scores to the images]

Two distinct papillary phenotypes are commonly seen during endoscopic examination of calcium oxalate (CaOx) stone formers. For example, the patients depicted in Fig. 1 were examined on the same day. Both patients presented with calcium oxalate stones: Micro CT analysis revealed that in the patient on the left, the stones consisted of 91% CaOx and 9% apatite, while in the patient on the right, they were composed of 76% CaOx and 24% apatite. Despite similar stone compositions, the appearance of their papillae differed significantly. The patient on the left exhibited mostly normal-looking papillae, except for the presence of Randall’s plaque. During the operation, seven papillae from this patient were assessed [17], with mean scores of 0.4 for plugging/dilated ducts, 0.7 for loss of papillary contour, and 0.9 for Randall’s plaque. Conversely, the patient on the right displayed ductal plugging in every papilla, with four papillae scoring means of 1.75 for plugging/dilated ducts, 1.5 for loss of contour, and 0.5 for Randall’s plaque. An average score of 1.75 for plugging means that *all papillae had plugs or dilated ducts*, with three of the four papillae having more than five yellow plaque deposits (plugs) or dilated ducts each [17].

**Figure 2:**
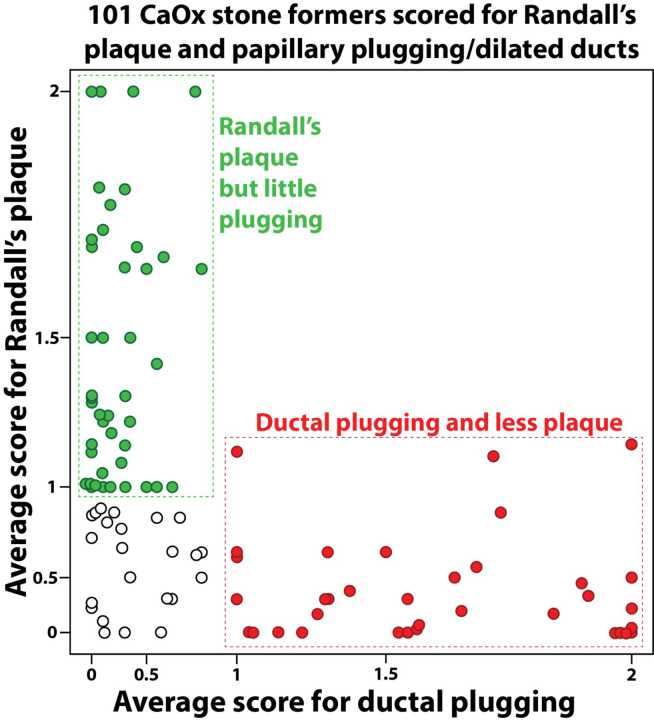
Idiopathic calcium oxalate (CaOx) stone formers tend to group into two papillary mineral patterns. Graph shows semi-quantitative grading of papillary mineral using endoscopic video in 101 CaOx stone formers. Axis scales are exponential. Note that a patient plugging score of 1.0 indicates that on average every papilla showed 1–5 plugs or dilated ducts; thus, a score <1.0 means that at least one papilla in that patient had no plugs or dilated ducts. In contrast, a Randall’s plaque score of 1.0 means that the average papilla was moderately covered with plaque, and a score of 0 means ‘mild’ coverage by plaque. Empty circles show patients with average scores <1 for both Randall’s plaque and plugging/dilated ducts (the Neither group in Table 1).

These two phenotypes were seen in our previous work [4], and we have now extended these observations to include 101 idiopathic CaOx patients. Papillary scoring revealed an inverse correlation between the amount of Randall’s plaque and the score for plugging/dilated ducts (Fig. 2, r^2^ = 0.34, *P* < 0.0001). Scores for plugging were positively correlated with loss of papilla mass (loss of contour, r^2^ = 0.27, *P* < 0.0001). Conversely, scores for Randall’s plaque were inversely correlated with loss of papilla mass (contour, r^2^ = 0.13, *P* = 0.0002).

Classifying these 101 patients into high-plaque, high-plugging, or neither, showed no distinctions in age, serum chemistries or 24-hour urine values (Table 1). There were more females in the high-plugging group (*P* = 0.04), and papillary pitting was more prevalent in both the high-plaque and high-plugging groups as compared with the patients who had low mineral scores. Overall, though, these patients were remarkably similar by most clinical measures.

### The severe nature of ductal mineral plugs

It is crucial to clarify that the plugging scored in this study represents grossly dilated collecting ducts so filled with mineral that they show up as yellow regions on the papilla tip [17, 19]. This plugging score also includes dilated collecting duct openings, indicating a ductal mineral plug that had since fallen out [17]. These dilated ducts and ductal plugs at the papilla tip are quite large (as much as 20 times larger in diameter than normal collecting ducts) [19], and are very different from the occasional plugged tubule that can be seen in normal kidneys higher up in the medulla, and which measure around 40 μm in diameter [20, 21]. Those mineral plugs seen in normal kidneys are smaller in diameter than a human hair, and so even if they were at the surface of the papilla, they would not be discernable by endoscope. The ductal plugs scored in these CaOx stone formers thus represent a severe pathology.

### Ductal plugging and the progressive loss of papilla tissue

**Figure 3:**
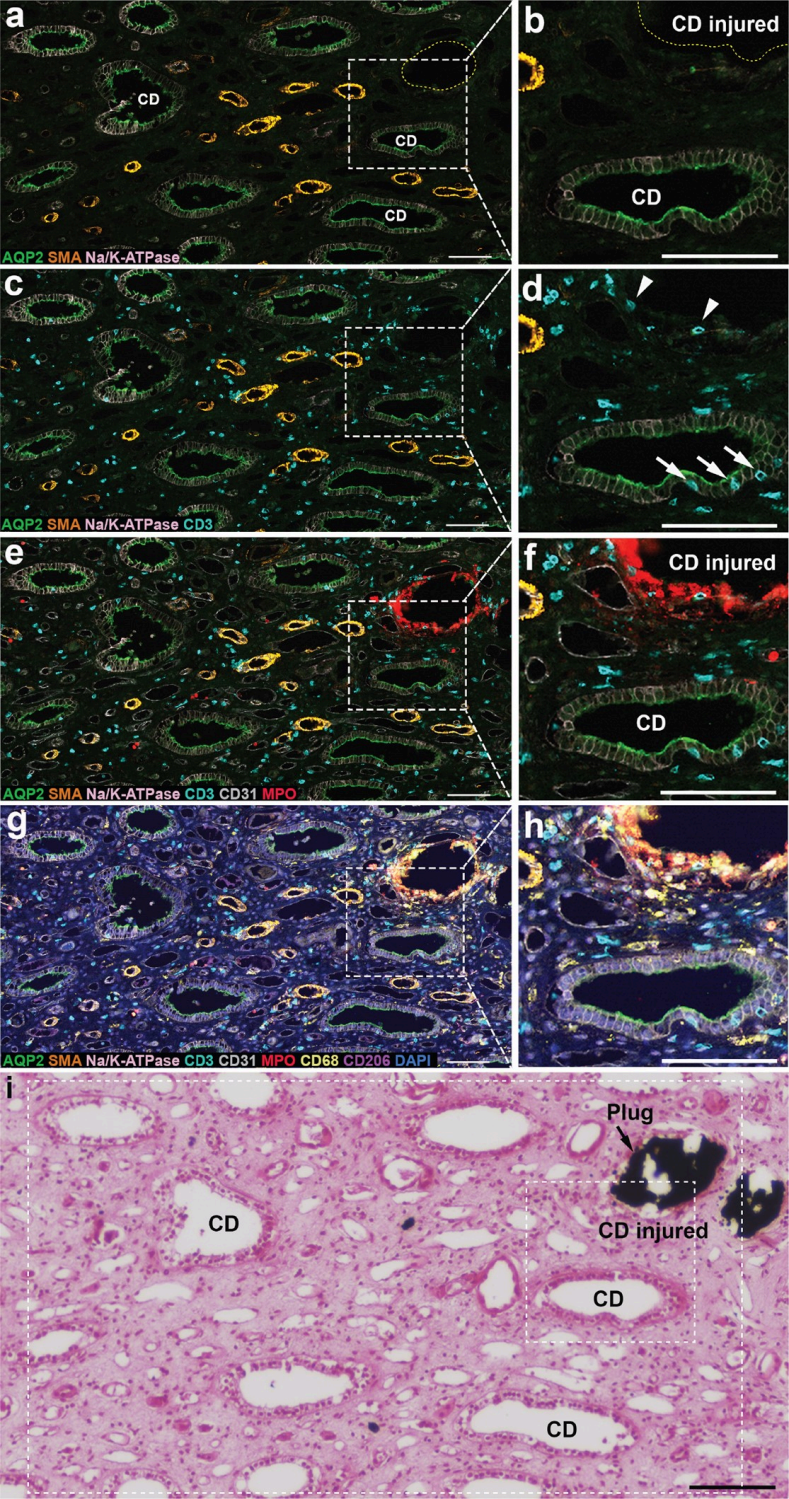
Multiplexed imaging on a biopsy from **a** CaOx stone former with ductal plugging. a. Antibody localization is shown for aquaporin 2 (green), alpha-SMA (orange), and NaK-ATPase (light pink). **b**. Blow-up showing injured collecting duct (which contained mineral, dashed yellow line), along with a normal appearing collecting duct. **c, d**. The same regions but with staining for T-cells revealed (cyan). Note T-cells invading the epithelium of the healthy collecting duct (arrows). **e, f**. Same regions but with visualization of CD31 (endothelium, light gray) and myeloperoxidase (neutrophils, red). Note coating of neutrophils around mineral plug. **g, h**. Visualization of macrophages (CD68, light yellow) and CD206 (marker of M2 macrophages, pink). Note that many macrophages also surround the mineral plug. i. Yasue stain of consecutive section, verifying the mineral plug in the collecting duct marked ‘injured’ in **b** and **f**. Dashed rectangles indicate approximate regions of panels a-h in this consecutive section. Scale bars are 100 μm.

The loss of papillary tissue with ductal plugging—measured by loss of papillary contour scores—is suggested by a hypothesis from 2005 in which the presence of a ductal mineral plug would incite destructive activity in nearby cells, so that the injury would progress through the papillary tissue [19]. Details of this published hypothesis included the injury of collecting duct cells by luminal crystals, death of those cells, expansion of the mineral plug so that it presses into the nearby tissue, and the resulting provocation of inflammation in tissues surrounding the plugged collecting duct [19]. Evidence for the last stages of this hypothesis is showing up in some newer work, as shown in Fig. 3, which shows a collecting duct adjacent to a ductal mineral plug. The collecting duct appears normal in structure, with the exception of the presence of T-cells migrating up between the principal cells (arrows in Fig. 3d). When lymphocytes are seen to invade renal tubular epithelium—a phenomenon called tubulitis—it is viewed as a sign of impending necrosis of the tubule [22].

Panels a and b in Fig. 3 show a healthy collecting duct (CD) lying close to a collecting duct that is filled with mineral and which shows very little staining for collecting duct cells (CD injured). In panels c and d, the healthy collecting duct is seen to have T-cells within its epithelium. (Note that this phenomenon of tubulitis in collecting ducts is difficult to detect without special stains.) There are also a few T-cells next to the mineral plug. Panels e and f add visualization of neutrophils, which are seen to completely surround the mineral plug. Panels g and h add visualization of macrophages, which are also surrounding the mineral plug, so that the mineral plug appears to be thoroughly enveloped by immune cells.

This focal investment of immune cells against the mineral plug is quite dramatic and points to a general increase in immune cell activity in the tissue. Note that the ductal plug in this case has not yet grown to a size that would press against neighboring tissues, but still there is T-cell activity attacking an adjacent collecting duct. This concept, in which a ductal plug would lead to destruction of nearby tissues, was first proposed for the profound papillary injury seen in papillary biopsies from brushite stone formers [19], but has also been proposed for other kinds of stone former in which ductal mineral plugs are also found [23]. These investigators also proposed that injury of a collecting duct epithelium might reduce its ability to acidify the tubular fluid and thus lead to the formation of a new calcium phosphate plug [19]. Thus, an initial ductal mineral plug would lead to injury to surrounding tubules which would then also become plugged with mineral, and the progression of tissue injury could spread through the papilla.

There may also be a role for reactive oxygen species in all of this, as crystals interacting with cells can induce the production of reactive oxygen species and so contribute to the inflammatory state [16, 24].

### Randall’s plaque and mild inflammation of the papilla tissue

**Figure 4:**
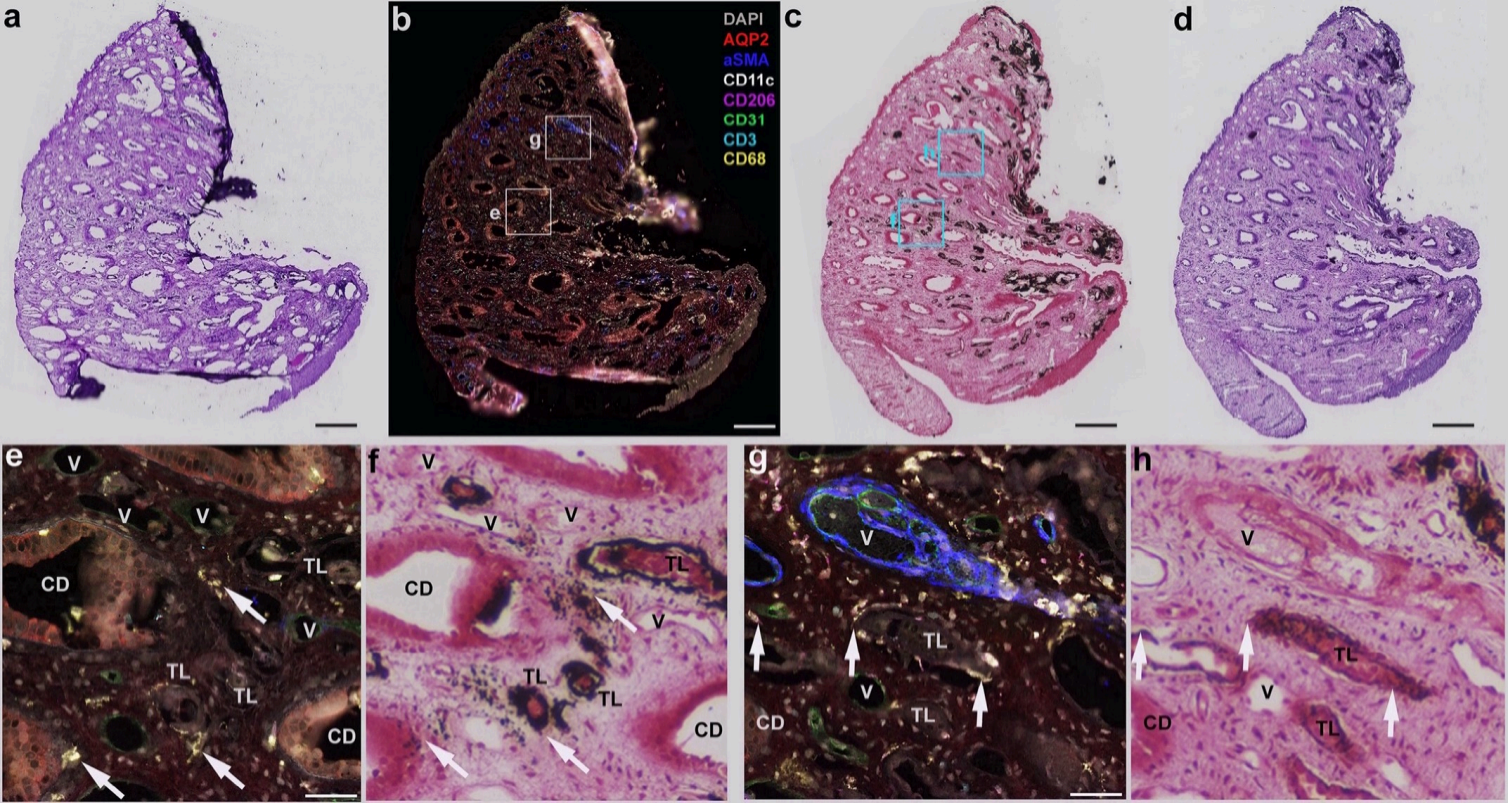
Papillary biopsy from a patient who had extensive deposition of Randall’s plaque, as seen by endoscopy. **a**. H&E staining of the multiplexed-imaged section *after*all the cycling of reagents and fluorescence imaging was complete; note remarkable preservation of the tissue. **b**. Multiplex image from that section: DAPI, stain for nuclei; AQP2, aquaporin-2 for collecting duct; aSMA, alpha-smooth muscle actin; CD11c, for antigen-presenting cells; CD206, for resident macrophages; CD31, labelling endothelium; CD3, labelling T-cells; and CD68, a general macrophage marker that is highly expressed in inflammatory states. **c, d**. Consecutive sections stained with Yasue and H&E, respectively. **e**. Higher power view of inset shown in b; arrows indicate CD68+ macrophages. **f**. Same region as e from Yasue stained section (**c**), showing that macrophages were associated with mineralization (Randall’s plaque, brown-to-black staining; note nearby thin limbs that have only dots of mineral). **g**. Higher power view of the other inset in **b**; V is a vessel surrounded by alpha-SMA-positive pericytes (blue), and arrows indicate CD68+ macrophages. **h**. Same region as **g**from Yasue stained section (**c**); again, locations of macrophages (arrows) are at regions of developing Randall’s plaque (brown; again, note variation in mineral deposition among thin limb segments). CD, collecting duct. TL, thin limb. Bars in a-d, 300 um; bars in e and g, 50 um.

Contrast this picture with that shown in Fig. 4, in which a biopsy from a CaOx stone former shows Randall’s plaque and no mineral plugs. The plaque regions—which are interstitial, initiating in the basement membranes of thin limbs [25]—have macrophages associated with them, but no tubulitis seen in any surrounding tubules. The association of Randall’s plaque with macrophages has been reported before [18]. An absence of tubular injury has been reported repeatedly for CaOx biopsies containing only Randall’s plaque [23, 25, 26].

Moreover, the inverse relationship of Randall’s plaque scores with scores for loss of papilla mass suggests that the presence of plaque is consistent with the overall health of the papillary tissue. Whereas the presence of ductal plugs of mineral is associated with loss of papilla tissue, an abundance of Randall’s plaque correlates with a healthier papilla appearance. When we see a patient with Randall’s plaque, the kidney often looks very healthy and the papillae show only Randall’s plaque and the very shallow erosion pits left from previous stone events [27].

### Hypothesis

**Figure 5:**
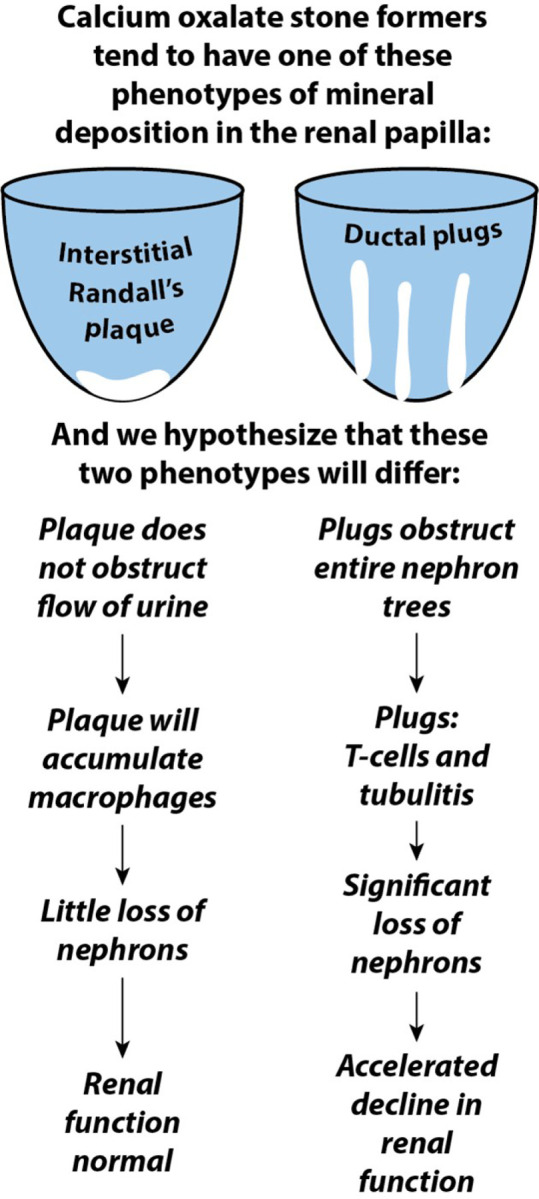
Extended hypotheses based on the observations that CaOx stone formers tend to have either Randall’s plaque or ductal plugging. The obstructive nature of ductal plugging suggests that it is more likely to lead to significant nephron loss. In addition, preliminary data on cellular inflammation suggests that ductal plugging is associated with an aggressive T-cell infiltrate that could also lead to nephron loss.

Putting these data together, we see that there are two phenotypes of CaOx stone former and it seems likely that long-term prognosis of these phenotypes may not be the same (Fig. 5). We hypothesize that the typical CaOx stone former making stones on Randall’s plaque has what is in the long term a disease that does not lead to destruction of papillary tissue, and which is unlikely to lead to loss of many nephrons. While this condition does result in a large number of stones [4] which are especially recurrent [2], the size of stones in this phenotype is relatively small [4]. The Randall’s plaque lesion is interstitial and the collecting ducts are free from luminal obstruction. The loss of calcified papillary tissue (Randall’s plaque) with a stone [4] must cause the breakage of loops of Henle, but with each stone lost only a few nephrons will be damaged. The immune cell infiltrate appears to consist mainly of macrophages collected near the region of interstitial mineral, with minimal T-cell activity [18]. All of this points to a form of stone disease that will not result in long-term degradation of renal function.

In contrast, CaOx stone formers who produce luminal mineral plugs in the papillae exhibit several features that could lead to renal function loss over time. The plugging of collecting ducts indicates profound obstruction of urine flow, with plugs visible at the surface of the papilla being significantly larger than tubular mineral plugs described in normal kidneys. Since each terminal collecting duct serves thousands of glomeruli, blockage of these could lead to loss of many nephrons and the resultant decline in renal function. Additionally, if active inflammation accompanies these massive tubular mineral plugs, further damage may occur to papillary tissue. This additional damage could include injury to surrounding collecting ducts, injury which can make them also susceptible to the precipitation of apatite in the lumen to plug up additional trees of nephrons. The visual observation of loss of papillary mass in kidneys with plugged collecting ducts aligns with this depiction of plugging being detrimental to papillary tissue. If papillary tissue is damaged in a way that destroys collecting ducts, renal cortical damage may also follow.

Currently, the only way to distinguish CaOx stone formers as being Randall’s plaque or plugging types is via endoscopic observation. Thus, testing these hypotheses of progression to chronic kidney disease will initially be limited to patient populations undergoing endoscopic stone removal. We predict that CaOx patients who show extensive ductal plugging in their papillae will also have, over time, an increased rate of decline in renal filtration function [28]. In contrast, CaOx patients without ductal plugging should show a decline in renal filtration function with age that is similar to non-stone formers.

## Conclusion

Our findings highlight the existence of distinct phenotypes among CaOx stone formers, characterized by either Randall’s plaque or ductal plugging in the papillae. These phenotypes exhibit dramatic differences in papillary appearance, nature of the inflammatory infiltrate, and in potential long-term prognosis. Ductal mineral plugs present as the more severe pathology, potentially leading to renal function decline over time. Further research is warranted to validate these observations and explore their implications for patient management and prognosis prediction in CaOx stone disease.

## Figures and Tables

**Table 1:** Clinical characteristics of patient groups shown in Fig. 2.

	High plugging scores	High Randall’s plaque scores	Neither
*N*	35	43	23
Age	52.6 ± 13.0	50.4 ± 16.6	50.7 ± 16.7
Sex (M/F)	11/24*	25/18	13/10
*Papillary plugging*	*1.57 ±0.36*	0.24 ± 0.24	0.37 ± 0.30
Papillary pitting	0.72 ± 0.59	0.94 ± 0.56	**0.31 ± 0.31** †
Loss of papillary contour	**0.46 ± 0.40** ‡	0.13 ± 0.22	0.23 ± 0.25
*Papillary Randall’s plaque*	0.35 ± 0.35	*1.40 ± 0.34*	0.55 ± 0.32
Serum Ca (mg/dL)	9.51 ± 0.58	9.42 ± 0.33	9.36 ± 0.35
Serum creatinine (mg/dL)	0.97 ± 0.29	0.93 ± 0.20	0.91 ± 0.16
Urine vol (24 h, L)	2.1 ± 0.6	2.0 ± 0.9	1.9 ± 0.9
Urine pH	6.1 ± 0.4	6.1 ± 0.4	6.0 ± 0.5
Urine citrate (mg/day)	568 ± 341	627 ± 355	724 ± 376
Urine Ca (mg/day)	271 ± 159	226 ± 113	237 ± 113
Urine oxalate (mg/day)	39 ± 18	39 ± 14	38 ± 13
Urine sodium (mmol/day)	168 ± 74	177 ± 77	166 ± 74
Supersaturation for CaOx	7.5 ± 3.6	6.9 ± 3.3	7.9 ± 4.2
Supersaturation for CaP	1.3 ± 1.1	1.3 ± 0.9	1.4 ± 1.1

*Italics* indicates scores used to distinguish groups.

* Chi square test yields *P* = 0.04 for sex distribution among these groups.

† Different from both high plugging and high Randall’s plaque group, *P* < 0.02 and *P*< 0.001, respectively.

‡ Different from both high Randall’s plaque and the ‘Neither’ group, *P* < 0.001 and *P* < 0.02, respectively.

## Data Availability

on request
